# A Consortium of *Pseudomonas aeruginosa* and *Trichoderma harzianum* for Improving Growth and Induced Biochemical Changes in *Fusarium* Wilt Infected Bananas

**DOI:** 10.21315/tlsr2021.32.1.2

**Published:** 2021-03-31

**Authors:** Clement Kiing Fook Wong, Dzarifah Zulperi, Noor Baity Saidi, Ganesan Vadamalai

**Affiliations:** 1 Department of Plant Protection, Faculty of Agriculture, Universiti Putra Malaysia, 43400 UPM Serdang, Selangor, Malaysia; 2 Department of Cell and Molecular Biology, Faculty of Biotechnology and Biomolecular Sciences, Universiti Putra Malaysia, 43400 UPM Serdang, Selangor, Malaysia

**Keywords:** Banana, Biocontrol Agents, Bioformulations, *Fusarium* Wilt, Pisang, Agen Kawalan Biologi, Bioformulasi, Layu *Fusarium*

## Abstract

*Fusarium* wilt of banana cannot be effectively controlled by current control strategies. The most virulent form that caused major losses in the banana production is *Fusarium oxysporum* f. sp. *cubense* Tropical Race 4 (Foc-TR4). Biocontrol of Foc-TR4 using microbial antagonists offers a sustainable and eco-friendly alternative. A consortium of biocontrol agents (BCAs), *Pseudomonas aeruginosa* DRB1 and *Trichoderma harzianum* CBF2 was formulated into pesta granules, talc powder, alginate beads and liquid bioformulations. Previous study indicated bioformulations containing both BCAs successfully reduced the disease severity of Foc-TR4. To date, the biocontrol mechanism and plant growth promoting (PGP) traits of a consortium of BCAs on infected bananas have not been explored. Therefore, the study was undertaken to investigate the effect of a consortium of DRB1 and CBF2 in the growth and biochemical changes of Foc-TR4 infected bananas. Results indicated pesta granules formulation produced bananas with higher biomass (fresh weight: 388.67 g), taller plants (80.95 cm) and larger leaves (length: 39.40 cm, width: 17.70 cm) than other bioformulations. Applying bioformulations generally produced plants with higher chlorophyll (392.59 μg/g FW–699.88 μg/g FW) and carotenoid contents (81.30 μg/g FW–120.01 μg/g FW) compared to pathogen treatment (chlorophyll: 325.96 μg/g FW, carotenoid: 71.98 μg/g FW) which indicated improved vegetative growth. Bioformulation-treated plants showed higher phenolic (49.58–93.85 μg/g FW) and proline contents (54.63 μg/g FW–89.61 μg/g FW) than Foc-TR4 treatment (phenolic: 46.45 μg/g FW, proline: 28.65 μg/g FW). The malondialdehylde (MDA) content was lower in bioformulation treatments (0.49 Nm/g FW–1.19 Nm/g FW) than Foc-TR4 treatment (3.66 Nm/g FW). The biochemical changes revealed that applying bioformulations has induced host defense response by increasing phenolic and proline contents which reduced root damage caused by Foc-TR4 resulting in lower MDA content. In conclusion, applying bioformulations containing microbial consortium is a promising method to improve growth and induce significant biochemical changes in bananas leading to the suppression of Foc-TR4.

## Abstract (ms)

Penyakit layu *Fusarium* pisang tidak boleh dikawal secara efektif dengan strategi kawalan semasa. *Fusarium oxysporum* f. sp. *cubense* Tropical Race 4 (Foc-TR4) adalah penyakit paling virulen yang menyebabkan kerugian besar dalam pengeluaran pisang. Kawalan biologi Foc-TR4 dengan penggunaan mikrob antagonis merupakan alternatif yang mampan dan mesra alam. Campuran agen-agen kawalan biologi (BCA), *Pseudomonas aeruginosa* DRB1 dan *Trichoderma harzianum* CBF2 telah diformulasikan kepada empat bioformulasi iaitu butiran pesta, serbuk talkum, butiran alginat dan formulasi cecair. Kajian terdahulu menunjukkan bioformulasi yang mengandungi kedua-dua BCA berjaya mengurangkan masalah penyakit pisang Foc-TR4. Setakat ini, mekanisme kawalan biologi dan ciri-ciri penggalak tumbesaran pokok bagi campuran BCA terhadap pokok pisang berpenyakit belum dikaji. Justeru, kajian ini dijalankan untuk mengkaji kesan campuran DRB1 dan CBF2 terhadap tumbesaran dan perubahan biokimia bagi pokok pisang yang dijangkiti Foc-TR4. Keputusan kajian menunjukkan formulasi butiran pesta menghasilkan pokok pisang yang tinggi (80.95 cm), mempunyai biomas tinggi (berat segar: 388.67 g) dan daun lebih besar (panjang: 39.40 cm, keluasan: 17.70 cm) berbanding formulasi lain. Aplikasi bioformulasi secara amnya menghasilkan pokok yang mempunyai kandungan klorofil (392.59 μg/g FW–699.88 μg/g FW) dan karotenoid (81.30 μg/g FW–120.01 μg/g FW) yang tinggi berbanding rawatan patogen (klorofil: 325.96 μg/g FW, karotenoid: 71.98 μg/g FW) dan keputusan ini adalah petunjuk kepada tumbesaran pokok yang baik. Pokok pisang yang dirawat dengan bioformulasi menunjukkan kandungan fenolik (49.58–93.85 μg/g FW) dan prolina (54.63 μg/g FW–89.61 μg/g FW) yang tinggi berbanding rawatan Foc-TR4 (fenolik: 46.45 μg/g FW, prolina: 28.65 μg/g FW). Kandungan malondialdehyde (MDA) juga lebih rendah dalam rawatan bioformulasi (0.49 Nm/g FW–1.19 Nm/g FW) berbanding rawatan Foc-TR4 (3.66 Nm/g FW). Perubahan biokimia dalam pokok pisang menunjukkan bahawa aplikasi bioformulasi telah mengakibatkan respons pertahanan pokok dengan meningkatkan kandungan fenolik, prolina serta mengurangkan kandungan MDA. Kandungan MDA rendah menunjukkan kerosakan akar pisang adalah minimum. Secara keseluruhannya, aplikasi bioformulasi yang mengandungi campuran mikrob dapat menggalakkan tumbesaran pokok dan mengaruhkan perubahan biokimia yang signifikan dalam kawalan penyakit Foc-TR4.

**Highlights**

- Four formulations (pesta granules, talc powder, alginate beads and liquid formulation) containing a consortium of *Pseudomonas aeruginosa* and *Trichoderma harzianum* were developed.
- The application of the pesta granule formulation improved the vegetative growth of bananas infected by *Fusarium oxysporum* f. sp. *cubense* Tropical Race 4 (Foc-TR4).
- The disease tolerance of banana plants against Foc-TR4 was improved as a result of induced defense response through the application of microbial consortium regardless of formulations.

## INTRODUCTION

*Fusarium* wilt of banana, caused by the fungal pathogen. *Fusarium oxysporum* f. sp. *cubense* (Foc) has hampered global and local banana productions. To date, the most virulent strain is the Tropical Race 4 (TR4). In Asia, the annual economic losses in Malaysia, Taiwan and Indonesia caused by Foc-TR4 were reported to be USD14.1 million, USD121 million and USD253.3 million, respectively (Food and Agriculture Organization Corporate Statistical Database [FAOSTAT] 2017). Currently, Foc-TR4 is found distributed in 19 of the 135 countries that produce bananas and its rapid spread has received tremendous global interest in seeking effective management methods (Zheng *et al*. 2018). The polycylic nature of Foc has complicated the development of long-term disease management strategies (Wibowo *et al*. 2013). In general, Foc is a difficult soil-borne fungus to control due to several reasons: (1) its persistent survival in soil for more than 20 years even in the absence of the banana host or within alternative host which do not show disease symptoms; (2) as a vascular pathogen that escapes non-systemic fungicides and non-endophytic biocontrol agents (BCAs); (3) easily spread through soil, planting materials, workers and farm machinery; and (4) the monoculture of popular banana varieties such as Cavendish (Bubici *et al*. 2019). Most current control measures including cultural practices, plant breeding, genetic engineering and chemical control of this pathogen were deemed ineffective and difficult to execute (Dita *et al*. 2018). Biological control is a sustainable alternative that utilises microbial antagonists or BCAs for the suppression of plant disease (Raza *et al*. 2016). Compared to other available methods, developing a biocontrol regime for Foc is not costly, it is sustainable and environmental-friendly method of managing Foc (Wang *et al*. 2017).

Biological control has gained attention in the integrated management of Foc due to the excessive input of pesticides which has caused environmental, health, safety and economic concerns (Liu & Prada 2018). The current biocontrol research emphasised largely on the use of a consortium of BCAs to achieve a more durable control over Foc-TR4 compared to the use of single strain (Raza *et al*. 2016). As each strain of compatible BCAs possesses unique antagonistic traits, the microbial consortium can be conveniently used to control a broader spectrum of pathogens (Thakkar & Saraf 2015). To further enhance the biocontrol efficacy of BCAs, they can be formulated into granular, powder and slurry liquid forms to maintain microbial viability and prolonged storage in adverse soil and environmental conditions (Klein *et al*. 2016). These tailor-made bioformulations of combined BCAs could thereby overcome inconsistencies in biocontrol efficacy by ensuring successful plant colonisation and improved microbial viability (Mercado-Blanco *et al*. 2018). The effectiveness of a consortium of bacterial or fungal BCAs in suppressing Foc while improving the vegetative growth and yield of banana plants was reported in various studies (Akila *et al*. 2011; Thangavelu & Gopi 2015a; 2015b). Furthermore, bioformulations of compatible BCA mixtures, containing nutrient or additive amendments, were found to improve the biocontrol efficacy of Foc (Keswani *et al*. 2016). Recently, the biocontrol efficacy of bio-formulated bacterial and fungal BCAs into pesta granules was also found to suppress Foc-TR4 under glasshouse condition but the impact of the microbial consortium on the vegetative growth of bananas were not evaluated (Wong *et al*. 2019). It was discovered that pesta granules contained flour as the major constituents with sucrose as additive which has contributed to the overall higher viability of BCAs thereby resulting in the suppression of Foc-TR4 (Wong *et al*. 2019). It was suggested that BCAs could have utilised the flour and sucrose to survive under harsh soil conditions.

A good biocontrol efficacy is often associated with the ability of BCAs in improving vegetative growth and host resistance towards plant pathogens which can be easily assessed using biochemical assays (Rais *et al*. 2017). BCAs that contain plant growth promoting (PGP) traits could improve plant growth during pathogen invasion by maintaining or increase the leaf chlorophyll and carotenoid contents so that photosynthetic activity is not compromised (Mishra *et al*. 2018). The application of BCAs also induced host plant defense by triggering host production of antimicrobial metabolites, cell wall hydrolytic enzymes and pathogenesis-related proteins to suppress plant pathogens (Mukherjee *et al*. 2012; Schulz-Bohm *et al*. 2017). In most studies, Foc-infected plants treated with single strain of BCA often demonstrated improved vegetative growth as a result of increased chlorophyll content whereas the production of antimicrobial metabolites such as phenolics and hydrolytic enzymes including chitinase and glucanase has led to disease suppression of Foc (Chen *et al*. 2008; Lobato *et al*. 2010; Fortunato *et al*. 2012; Jain *et al*. 2012; Mishra *et al*. 2018). However, the effect of a consortium of BCAs on the biochemical changes in Foc-TR4 infected banana plants has yet to be investigated.

Previously, a total of four bioformulations (pesta granules, talc powder, alginate beads and liquid formulation) containing a compatible pair of *Pseudomonas aeruginosa* DRB1 and *Trichoderma harzianum* CBF2 were developed and their biocontrol efficacy against Foc-TR4 was determined (Wong *et al*. 2019). Both BCAs were chosen since they were commonly known as good candidates of microbial antagonists (Pandey *et al*. 2016; Cabanás *et al*. 2018). Most biocontrol studies also reported that Foc was effectively controlled up to 79% by using *Pseudomonas* spp. and up to 70% by *Trichoderma* spp. (Bubici *et al*. 2019). Nonetheless, their effect as a microbial consortium on plant growth and host biochemical changes was left unexplored. Hence, this study sought to investigate the impact of the same bioformulations containing a consortium of DRB1 and CBF2 on the vegetative growth and biochemical changes of banana plants that led to the suppression of Foc-TR4.

## MATERIALS AND METHODS

### Microbial Strains and Plant Materials

*Pseudomonas aeruginosa* DRB1, *T. harzianum* CBF2 and Foc-TR4 (VCG 01213/16) were obtained from Laboratory of Microbial Biological Control, Department of Plant Protection, Universiti Putra Malaysia. A 48-h DRB1 suspension culture was adjusted to 2 × 10^10^ cfu/mL whereas spore suspension from 7-day-old CBF2 culture was adjusted to 4 × 10^9^ cfu/mL. A consortium of DRB1 and CBF2 was added to the dry and liquid formulations at a ratio of 1:1. Both BCAs were tested to be compatible to each other as reported earlier in previous findings (Wong *et al*. 2019). Two-month-old *Musa acuminata* AAA Berangan seedlings, which are susceptible to Foc-TR4, were used in glasshouse study.

### Determination of PGP Properties of BCAs

Indole acetic acid (IAA) production was determined based on the method of Brick *et al*. (1991). A colony of bacterial BCA and a plug of fungal BCA were inoculated onto nutrient broth (NB) and potato dextrose broth (PDB), respectively. Both bacterial and fungal cultures were incubated for five and seven days, respectively. The suspension culture was centrifuged at 12 000 xg for 10 min. One mL of supernatant was added to 50 μL of phosphoric acid and 2 mL of IAA reagent (1 mL of 0.5 M of FeCl_3_·6H_2_O and 50 mL of 35% (v/v) of perchloric acid). The mixture was incubated for 30 min at room temperature and absorbance was read at 530 nm. The IAA hormone was used as a standard. Results were expressed as μg IAA/mL.

The phosphate solubilising activity for each BCA was determined using the National Botanical Research Institute’s Phosphate (NBRIP) growth medium (Nautiyal 1999). A single colony of bacteria and a fungal mycelia plug were inoculated respectively into the NBRIP broth and incubated for five days at room temperature on an orbital shaker at 150 rpm. The suspension cultures were centrifuged at 10 000 xg for 10 min. A volume of 750 μL of supernatant was added to 750 μL of reagent comprising of 1.5% (w/v) (NH_4_)6Mo_7_O_24_, 5.5% (v/v) sulphuric acid and 2.7% (w/v) Fe_2_SO_4_ solution. The mixture was allowed to incubate for 5 min at room temperature before absorbance was read at 600 nm. Ammonium phosphate was used as a standard. The amount of phosphate in the medium was expressed as μg phosphate/mL (Fiske & Subbarow 1925).

The nitrogen fixing ability of the bacterial isolate was assessed according to Baldani *et al*. (2014). One milliliter of an overnight bacterial culture was added to 9 mL of nitrogen free bromothymol blue (NFb) semi-solid medium. The mixture was incubated at room temperature for seven days on an orbital shaker at 150 rpm. The formation of blue solution indicated the presence of nitrogen fixation activity.

To detect ammonia production, 1 mL of an overnight bacterial culture and a seven-day-old fungal spore suspension culture were inoculated into 9 mL of peptone broth (10 g/L peptone, 5 g/L NaCl, pH 7.0). The bacterial and fungal BCAs were incubated for three and five days, respectively. A volume of 500 μL of Nessler’s reagent was added to the culture to observe the formation of brown or red precipitate (Cappuccino & Sherman 1992).

Siderophore production of each BCA isolates was determined using the method of Alexander and Zuberer (1991). This assay was a combination of four main components, namely solution 1, 2, 3 and 4. For solution 1, 10 mL of 1 mM FeCl_3_·6H_2_O was added to 50 mL of 1.21 g/L chrome azurol S (CAS). The resulting dark purple solution was added to 40 mL of 1.821 g/L CTAB. For solution 2, 3 g MOPS was added to a 750 mL of salt solution comprising of 1 g NH_4_Cl, 0.5 g NaCl, 0.3 g K_2_HPO_4_ and 15 g agar. The pH was adjusted to 6.8 and distilled water was added to 800 mL. For solution 3, a 75 mL mixture consisting of 2 g glucose, 2 g mannitol, 493 mg MgSO_4_·7H_2_O, 11 mg CaCl_2_, 1.17 mg MnSO_4_·H_2_O, 1.4 mg H_3_BO_3_, 0.04 mg CuSO_4_·5H_2_O, 1.2 mg ZnSO_4_·7H_2_O and 1.0 mg NaMoO_3_ was prepared. All three solutions was autoclaved separately and cooled at room temperature. Solution 2 was added to solution 3. The mixture was added with filter-sterilised 30 mL casamino acid 10% (w/v) and this mixture was named as solution 4. Lastly, solution 1 was added to solution 4 to form a 1 L siderophore agar medium.

### Development of Bioformulations

A total of four bioformulations were developed. Pesta granules were formulated based on Mejri *et al*. (2013) with slight modifications. A mixture of 200 g kaolin and 800 g wheat flour was autoclaved for 20 mins at 121 psi and 200 g of sucrose was added after that. The mixture was added with an equal volume of 200 mL DRB1 and 200 mL CBF2 and mixed aseptically until it formed a cohesive dough. The dough was flattened until 1 mm thick and air dried for at least 24 h. The dried sheets were broken into smaller pieces. They were passed through a 2 mm sieve to obtain uniform granule sizes.

Talc powder formulation consisting of 1 000 g talc, 10 g carboxymethylcellulose (CMC), 15 g CaCO_3_ and 600 g kaolin was prepared according to Thangavelu and Gopi (2015a). The mixture was autoclaved consecutively for two times for 30 min at 121 psi. An equal volume of 400 mL DRB1 and 400 mL CBF2 were added to the mixture and mixed aseptically. The mixture was spread thinly on aluminum foil and air dried for at least 24 h in a laminar air flow. The dried sheets were blended into fine powder using a kitchen blender.

Alginate beads were formulated according to Zohar-Perez *et al*. (2003). The 1 L alginate mixture containing 4% (w/v) sodium alginate, 0.5% (w/v) kaolin, 60% (v/v) glycerol and distilled water was prepared and autoclaved for 20 min at 121 psi. An equal volume of 250 mL DRB1 and 250 mL CBF2 were added to the mixture. By using a 1 mm sterile syringe, the mixture was slowly dropped into a sterile 0.1 mM CaCl_2_ solution. The beads were left in the solution for an hour. They were filtered using sterile cheese cloth and washed three times with sterile distilled water. The beads were air dried in a laminar air flow for 24 h and stored in air-tight containers.

Based on Manikandan *et al*. (2010) research, the liquid formulation was prepared by adding 2% (v/v) filter sterilised glycerol to a microbial suspension containing equal volume of 500 mL DRB1 and 500 mL CBF2. The mixture was mixed well before using.

### Glasshouse Experiment Setup

The experiment was conducted in a glasshouse at Ladang 2, Universiti Putra Malaysia, Serdang, Selangor (3°02′30.7″N, 101°42′15.9″E) from January to March 2018. The experiment was carried out in a randomised complete block design (RCBD) with 15 replications for seven treatments. The experiment was repeated twice (Table 1). The steam-sterilised compost soil was pre-treated with bioformulations and benomyl fungicide before bananas were planted. A total of 50 g of dry bioformulation was added to 2 kg of soil (Zacky & Ting 2015). For liquid bioformulation, a volume of 100 mL was added to the same amount of soil (Pastrana *et al*. 2016). The fungicide, benomyl was applied as soil drench (100 mL) at a rate of 25 μg/mL active ingredient (AI) as recommended by Nel *et al.* (2007). The treated soil was moistened and left incubated at glasshouse for seven days. Susceptible Berangan plantlets were infected with Foc-TR4 using root dip method and planted directly (Purwati *et al.* 2008). Plants were watered daily and were fertilised fortnightly with 1 g of NPK blue (15:15:15) pellets. Plants treated with sterile distilled water and Foc-TR4 treatment served as negative and positive controls respectively.

### Plant Growth Assessment

The growth parameters were assessed after 84 days after inoculation (DAI). Ten whole plants were randomly removed and washed under running tap water. Plants were air-dried before fresh weight was measured. To measure dry weight, whole plants were dried continuously at 70°C until constant weight was recorded. Plant height was measured from the base of the corm until the tip of the longest leave. To determine the number of leaves, only fully expanded leaves were included. Leaf width and length were determined from the third leaf of the plant. The diameters of pseudostem and corm were measured using a caliper.

### Biochemical Assays

All biochemical assays were conducted after 84 DAI. About 0.5 g of leaf samples were ground using a mortar and pestle in liquid nitrogen. Ten milliliters of 80% (v/v) acetone was added to the powdered samples. Calcium carbonate was later added to the extracts at 0.5 mg to prevent the formation of pheophytin. The extracts were centrifuged at 12 000 xg at 4°C for 10 min. The supernatant was used to measure absorbance at 470 nm, 645 nm and 663 nm using a UV-Vis spectrophotometer (Multiskan Go, Thermofisher, USA). The total chlorophyll and carotenoid content was determined according to Arnon (1949) and Lichtenthaler and Buschmann (2001).

To determine total chlorophyll content:

$$\text{Total chlorophyll content}\;(\mu \text{g}/\text{g FW})=(20.2\times {\text{Abs}}_{645})+(8.02\times {\text{Abs}}_{663})\times \left(\frac{v}{1000}\times w\right)$$

where Abs = absorbance; *v* = final volume of solution and *w* = weight of sample.

To determine total carotenoid content:

$$\begin{array}{l} {\text{C}}_{\text{a}}\;(\mu \text{g}/\text{mL})=12.25\;{\text{Abs}}_{663}-2.79\;{\text{Abs}}_{645}\\ {\text{C}}_{\text{b}}\;(\mu \text{g}/\text{mL})=21.50\;{\text{Abs}}_{645}-5.10\;{\text{Abs}}_{663}\\ \text{Total carotenoid content}(\mu \text{g}/\text{g FW})=(1000\;{\text{Abs}}_{470}-1.82{\text{C}}_{\text{a}}-85.02{\text{C}}_{\text{b}})/198\times \left(\frac{v}{1000}\times w\right) \end{array}$$

where C_a_ = chlorophyll A; C_b_ = chlorophyll B; Abs = absorbance; *v* = final volume of solution and *w* = weight of sample.

The phenolic content of root samples was determined according to Fortunato *et al*. (2012). A 100 mg of root was crushed into fine powder using liquid nitrogen and 1.5 mL of 80% (w/v) methanol was added. The mixture was left incubated overnight at room temperature. The mixture was centrifuged at 12 000 xg for 5 min. A volume of 150 μL of methanol extract was added to 150 μL of Folin-Ciocalteu’s reagent and incubated for 5 min at room temperature. A volume of 150 μL of 1 M NaCO_3_ was added and incubated for another 10 min and later 1 mL of distilled water was added. The mixture was read at 725 nm. Gallic acid was used as a standard. Results obtained was expressed as μg gallic acid/g FW.

The centrifuged pellet obtained from above was used for the determination of lignin content (Fortunato *et al*. 2012). The pellet was homogenised with sterile distilled water and centrifuged at 12 000 xg for 10 min. The supernatant was discarded and samples were dried at 65°C overnight. A volume of 1.5 mL of acidified thioglycolic acid (1:10 of thioglycolic acid and 2 N HCl) was added to the dry residue and mixed gently. The mixture was placed in 95°C for 4 h. The mixture was cooled on ice for 10 min and centrifuged at the same conditions. The supernatant was discarded and 1.5 mL of 0.5 N NaOH was added. The mixture was left incubated overnight at room temperature and centrifuged on the next day, at the same condition. The supernatant was added with 200 μL of concentrated HCl and transferred to 4°C for 4 h to precipitate the lignin-thioglycolic acid (LTGA) derivatives. The precipitate was centrifuged at the same condition and the supernatant was discarded. The pellet was dissolved in 2 mL of 0.5 N NaOH and measured at 280 nm. Alkali lignin was used as a standard. Results obtained were expressed as μg LTGA/g FW.

The method of Bates *et al*. (1973) was used to determine the proline content in root samples. A 100 mg root sample was homongenised in 3% (w/v) of sulfosalicylic acid and centrifuged at 10 000 xg for 15 min at 4°C. A volume of 2 mL supernatant was added to 2 mL acid ninhydrin (1.25 g ninhydrin in 30 mL glacial acetic acid and 20 mL 6 M phosphoric acid) and 2 mL of glacial acetic acid. The mixture was incubated at 95°C for 1 h and incubated on ice for 2 min. A volume of 4 mL toluene was added and the mixture was agitated for 1 min. The toluene extract containing proline was measured at 520 nm. Proline was used as a standard. Results obtained was expressed as μM proline/g FW.

Malondialdehyde (MDA) content in roots was determined based on Heath and Packer (1968). A 100 mg root sample was homogenised in 4 mL of 1% (w/v) trichloroacetic acid (TCA) and centrifuged at 10 000 xg for 10 min at 4°C. A volume of 1 mL supernatant was added to 4 mL of 20% (w/v) TCA containing 0.5% (w/v) 2-thiobarbituric acid (TBA). The mixture was heated at 95°C for 30 min and cooled on ice for 2 min. The absorbance was read at 532 nm and 600 nm, respectively. The MDA content was calculated using the Beer-Lambert’s law with extinction coefficient of 155 mm^−1^.

### Statistical Analysis

The data obtained were analysed by one-way ANOVA. Mean values were compared by Duncan’s multiple range test at *p* ≤ 0.05 significance level using SAS version 9.4 (SAS Inc., USA).

## RESULTS AND DISCUSSION

### PGP Properties of BCAs

Based on Table 2, *P. aeruginosa* DRB1 produced 4.1 μg/mL of the hormone IAA while no IAA production was detected in *T. harzianum* CBF2. IAA is a phytohormone that enhances root growth for better uptake of soil nutrients and water. The synthesis of IAA by PGP bacteria and fungi occurs in a series of transamination and decarboxylation reactions of an amino acid precursor, tryptophan, commonly found in root exudates (Kuan *et al*. 2016). In this study, *P. aeruginosa* DRB1 produced 4.1 μg/mL of IAA which was in agreement with Khare and Arora (2010) whereby *P. aeruginosa* strain TO3 produced similar amount of IAA (4.8 μg/mL). In another study, *P. aeruginosa* strain AL2-14B produced higher amount of IAA (114.79 μg/mL) compared to *P. aeruginosa* DRB1 (Devi *et al*. 2017). The amount of IAA produced *in-vitro* could be dependent on the strains, the amount of tryptophan and incubation time.

Phosphate (P) solubilisation was observed in both *P. aeruginosa* DRB1 and *T. harzianum* CBF2 with P solubilising activity recorded at 33.7 mg/L and 2273.0 mg/L, respectively (Table 2). P is the second most important plant macronutrients after nitrogen (N) but P usually occurs in insoluble forms making it impossible to be absorbed by plants (Alori *et al.* 2017). BCAs from the genus *Pseudomonas* was reported to secrete low molecular weight organic acid such as gluconic acid that chelates specifically to P, making it available for root uptake (Oteino *et al.* 2015). The genus *Trichoderma*, on the other hand, produced phosphatase or phytase enzymes to solubilise P so that plants can easily acquire P through their root system (Alori *et al.* 2017). In other words, both DRB1 and CBF2 could have produced specific organic acids or enzymes that facilitated P solubilisation which require further validation in future study.

**Figure 1:**
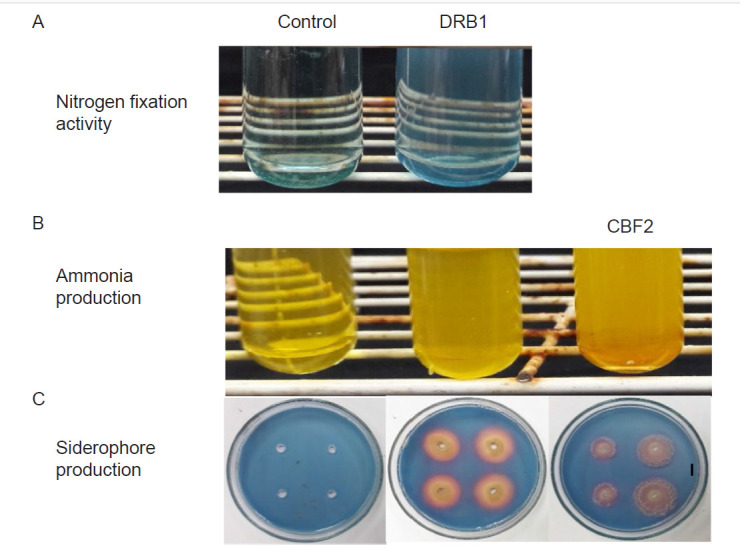
PGP properties of DRB1 and CBF2 as indicated by the formation of halo zones and changes of colour in medium and filter paper. Bar = 5 mm.

*P. aeruginosa* DRB1 exhibited nitrogen fixing ability by turning the Nfb semi-solid broth from green (pH 7.0) to blue (Fig. 1A, Table 2). Similar to P, atmospheric N cannot be readily uptake by plants until it is converted into ammonia through biological N fixation by soil microbes (Roychowdhury *et al*. 2017). The genus *Pseudomonas* is commonly used as biofertilisers due to its N fixing activity which could improve overall N uptake and boost plant growth (Li *et al*. 2017). On the other hand, both *P. aeruginosa* DRB1 and *T. harzianum* CBF2 produced ammonia by changing the colour of peptone water from yellow to dark yellow (Fig. 1B, Table 2). Ammonia production is an important PGP trait since plants can easily absorb ammonium for plant growth besides nitrate (Goswami *et al*. 2013). Siderophore production was also observed in *P. aeruginosa* DRB1 and *T. harzianum* CBF2 with a colour changes of the CAS medium from blue to orange (Fig. 1C, Table 2). *Pseudomonas* and *Trichoderma* were reported to produce high iron (Fe)-affinity siderophores to chelate iron from the environment and thus, allowing plants to sequester iron from these microbial siderophores for growth (Zeilinger *et al*. 2016). The genus *Pseudomonas* produced siderophores such as pseudobactin or pyoverdin under iron limiting conditions for plant uptake (Jan *et al*. 2011). Coprogen, ferrirocin and harzianic acid were produced by the genus *Trichoderma* and they had similar functions as bacterial siderophores (Vinale *et al*. 2013, Zeilinger *et al*. 2016).

### Improved Growth of Bananas

**Figure 2:**
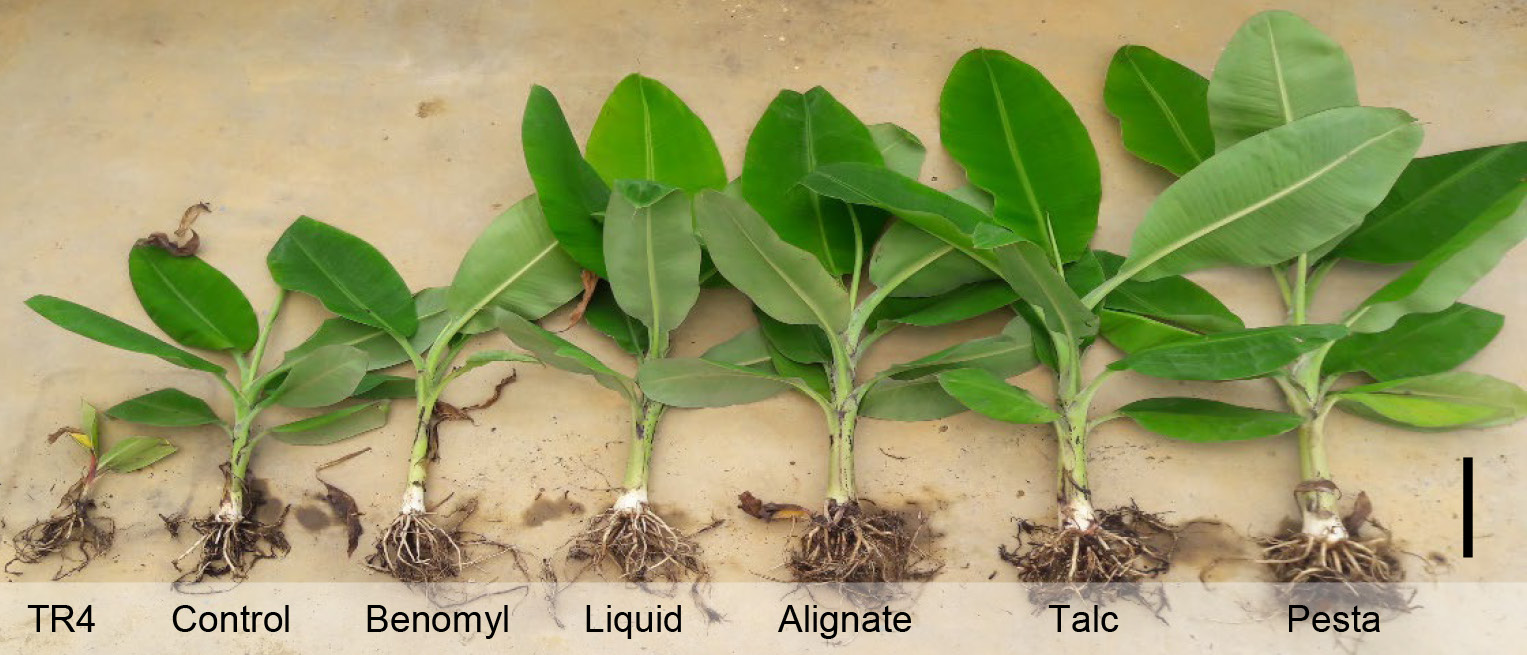
The application of bioformulation improved overall growth of bananas compared to Foc-TR4, control (without pathogen inoculation) and benomyl treatments. Bar = 10cm.

**Figure 3:**
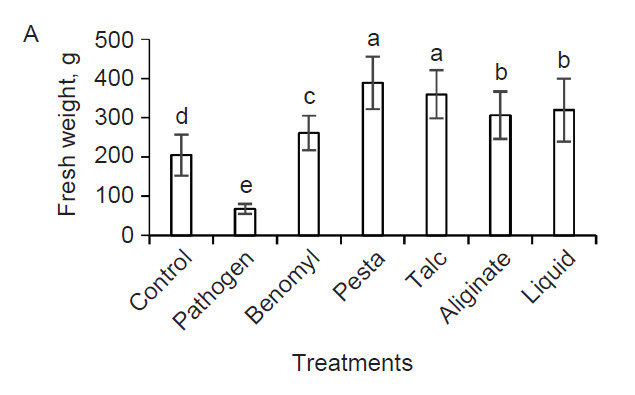

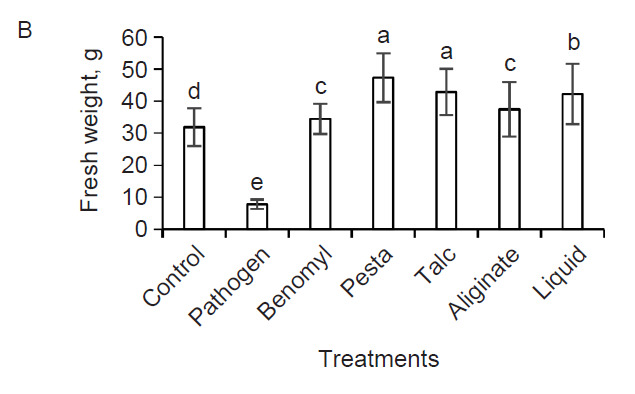

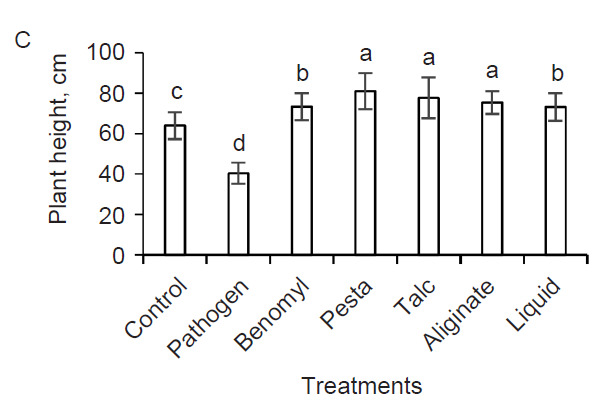

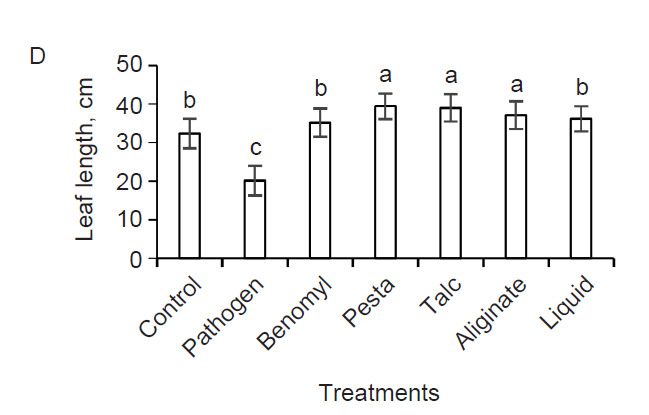

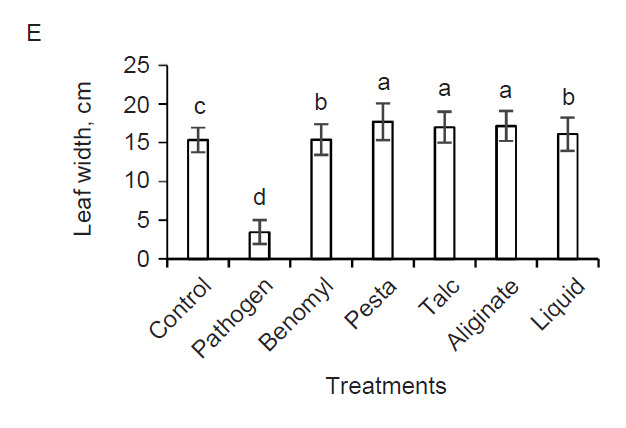

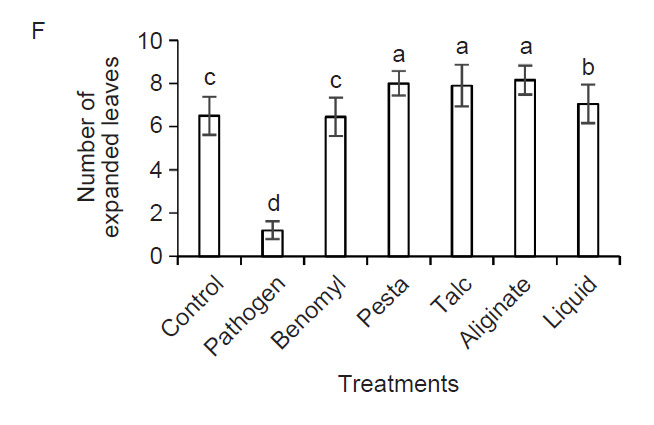

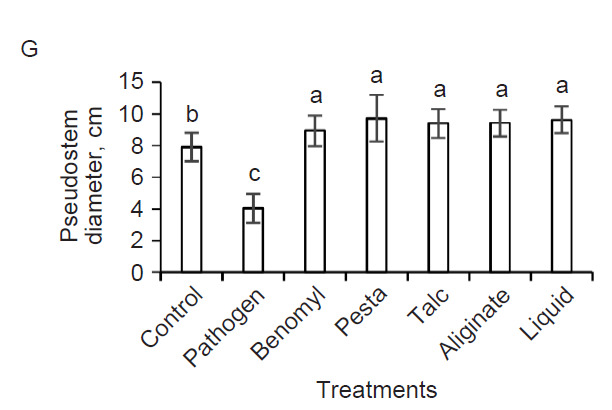

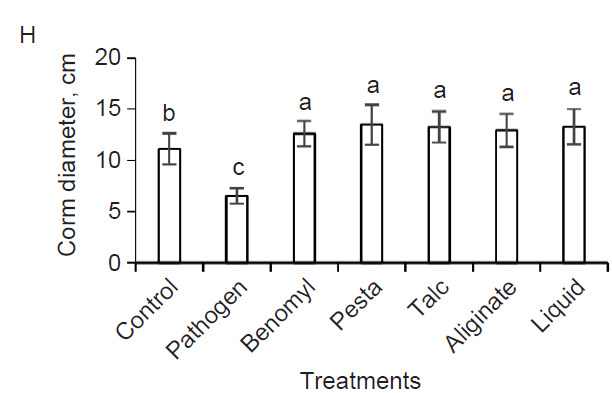
Changes of growth parameters of Foc-TR4 infected banana plants treated with benomyl and bioformulations after 84 DAI. (A) Fresh weight, (B) dry weight, (C) plant height, (D) leaf length, (E) leaf width, (F) number of expanded leaves, (G) pseudostem diameter and (H) corm diameter. Data represents mean ± SD of three replications. Different letters indicate the values are significant different (*p* ≤ 0.05).

After 84 DAI, the application of bioformulation containing a consortium of *P. aeruginosa* DRB1 and *T. harzianum* CBF2 has improved the overall physiological growth of bananas when challenged with Foc-TR4 as compared to bananas treated with benomyl, Foc-TR4 or without treatments (Fig. 2). In terms of biomass, plants treated with pesta granules have the highest fresh weight (388.67 g) and dry weight (47.33 g), followed by talc powder (359.33 g and 42.89 g), liquid formulation (319.33 g and 42.25 g) and alginate beads (306.00 g and 37.46 g), respectively (Figs. 3A and 3B). The application of pesta granules (80.95 cm) and talc powder (77.65 cm) also resulted in taller plants which was followed by alginate beads (75.35 cm) and liquid formulation (73.10 cm) (Fig. 3C). The leaf length and width were significantly higher in plants treated with pesta granules (39.40 cm and 17.70 cm), talc powder (39.00 cm and 17.00 cm) and alginate beads (37.15 cm and 17.15 cm) than liquid formulation (36.15 cm and 16.10 cm) (Figs. 3D and 3E). Compared to control, benomyl and Foc-TR4 treatments, there was no significant difference observed in the number of expanded leaves, pseudostem and corm diameter regardless of bioformulations used (Figs. 3F, 3G and 3H).

The PGP properties of *P. aeruginosa* DRB1 and *T. harzianum* CBF2 were determined initially in the in-vitro plate assays. PGP fungi and bacteria were known to be involved in improving plant growth by producing phytohormones such as IAA for stimulation of root growth in order to enhance nutrient and water uptake (Kuan *et al.* 2016). Moreover, PGP bacterial stimulated plant development by increasing plant height and overall leaf area resulting in improved plant biomass (Backer *et al.* 2018). PGP microbes are also well-known as good solubilisers of insoluble minerals such as phosphate and nitrogen so that plants can readily absorb them for vegetative growth (Smith *et al.* 2015). A consortium of *P. aeruginosa* DRB1 and *T. harzianum* CBF2 formulated into pesta granules was also found to suppress the wilting severity of Foc-TR4 on Berangan plants under glasshouse condition (Wong *et al.* 2019). The pesta granules consisted mostly of flour with additives such as sucrose which could serve as carbon sources for BCAs to grow and survive in the rhizosphere and roots of banana plants. As a result, the microbial viability of *P. aeruginosa* DRB1 and *T. harzianum* CBF2 were found to be higher in banana plants applied with pesta granules leading to better suppression of Foc-TR4 compared to other bioformulations (Wong *et al*. 2019). It is suggested that better viability of BCAs could have a better competition against Foc-TR4 which allows the BCAs to exert their PGP effects on bananas during the course of Foc-TR4 invasion.

### Induced Biochemical Changes in Bananas

**Figure 4:**
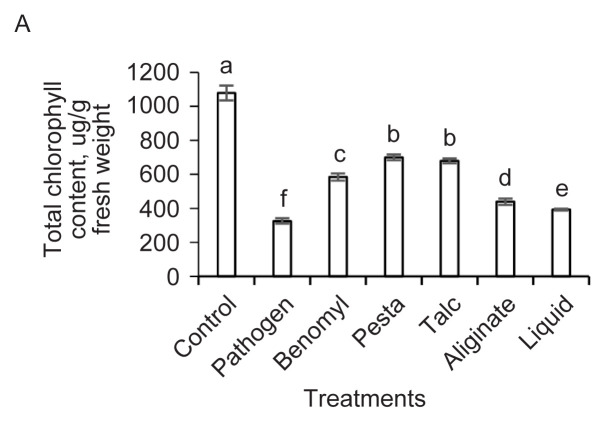

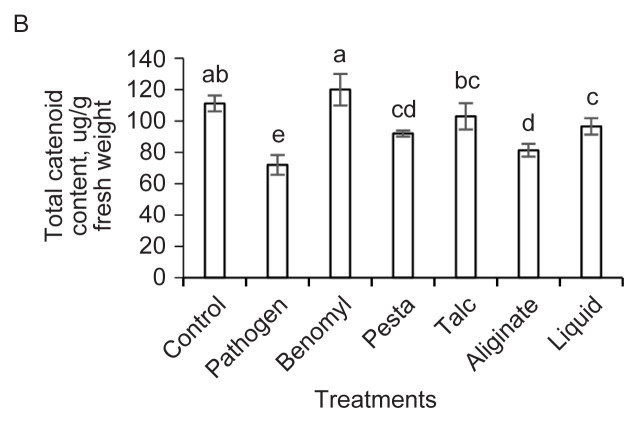

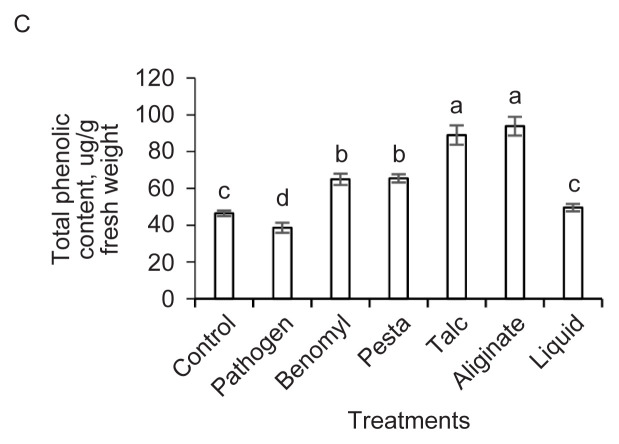

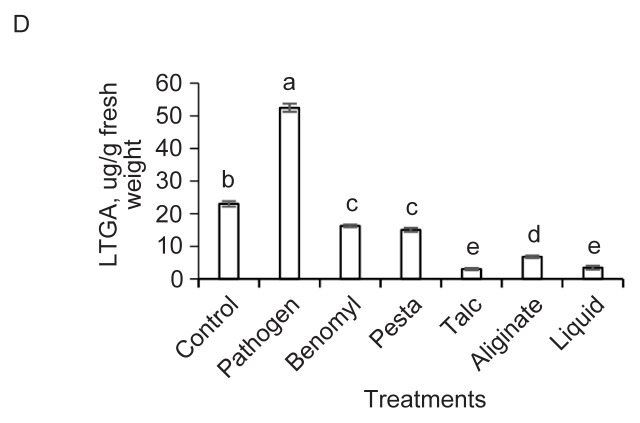

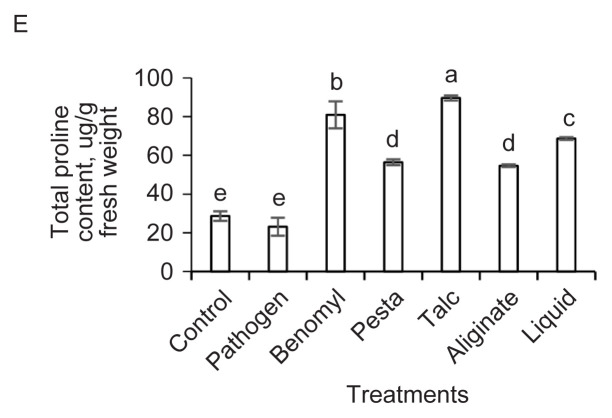

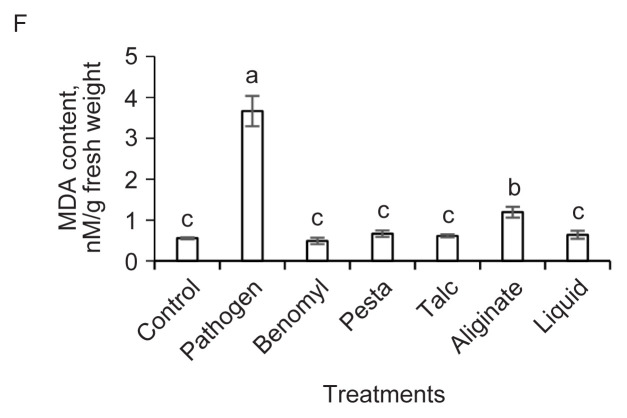
Biochemical changes of (A) chlorophyll, (B) carotenoid, (C) phenolic, (D) LTGA lignin derivative, (E) proline and (F) MDA in Foc-TR4 infected banana roots after 84 DAI. Data represents mean ± SD of three replications. Different letters indicate the values are significant different (*p* ≤ 0.05).

A good consortium of BCAs is attributed by their ability in sustaining or improving vegetative growth while at the same time, improving host resistance towards plant pathogens by inducing biochemical changes in host plants. The chlorophyll content in control treatment was the highest (1078.82 μg/g FW) compared to benomyl (584.16 μg/g FW) and bioformulation-treated plants (392.59 to 699.88 μg/g FW). Foc-TR4 treated plants contained the lowest chlorophyll content (325.96 μg/g FW) due to severe wilting symptoms (Fig. 4A). During early pathogen invasion, stomatal closure is an immediate response that limits the photosynthetic rate of plants (Nogués *et al.* 2002). As infection progresses, reduced CO_2_ assimilation leads to disruption in the host photosynthetic system, chloroplast function and eventually wilting of leaves (Dong *et al.* 2016). To minimise the extent of wilting, the application of BCAs were found to improve the chlorophyll content and maintained the photosynthetic capacity of plants during pathogen invasion (Bueno *et al.* 2017). The use of bioformulations containing *P. aeruginosa* DRB1 and *T. harzianum* CBF2 resulted in higher chlorophyll content in banana leaves than Foc-TR4 treatment which was in agreement with previous studies. *Pseudomonas* increased the formation of thylakoid membrane structures in leaf chloroplasts and chlorophyll content in leaves of *Brasicca napus* inoculated with the fungal pathogen *S. sclerotiorum* (Duke *et al.* 2017). *Trichoderma* also maintained the photosynthetic apparatus while enhancing the CO_2_ assimilation in rice leaves infected with the fungal scald disease (Bueno *et al.* 2017). Compared to control treatment, bioformulation-treated bananas contained lower chlorophyll content. Similar to other reports, BCA-treated plants contained lower or showed no significant difference in chlorophyll content than control treatment (Patel & Saraf 2017a; 2017b). The overall improved plant growth in bioformulation-treated Berangan could have contributed to lower chlorophyll content as energy was diverted for vegetative growth under stress conditions (Teh *et al.* 2015).

Banana plants treated with benomyl and bioformulations contained higher carotenoid content in the leaves (Fig. 4B). The highest carotenoid content was found in benomyl treatment (120.01 μg/g FW), followed by talc powder (103.00 μg/g FW), liquid formulation (96.52 μg/g FW), pesta granules (92.02 μg/g FW) and alginate beads (81.30 μg/g FW) as compared to Foc-TR4 treatment (71.98 μg/g FW). Beneficial microbial inoculants have been studied for their role in the accumulation of antioxidants such as carotenoids in plants during biotic and abiotic stress conditions (Alori & Babalola 2018). Carotenoids are accessory pigments in plants that stabilises the membrane of chloroplasts and thylakoid membranes from being susceptible to lipid peroxidation due to formation of reactive oxygen species (ROS) caused by plant pathogens (Lobato *et al*. 2010; Taïbi *et al*. 2016). The accumulation of carotenoid content in bioformulation-treated bananas could have resulted in higher antioxidative potential against ROS generated by Foc-TR4 during the course of host invasion, which might have protected the chloroplast from degradation and thus improving the photosynthetic capacity of the plants. Similar observation was reported in which the application of a consortium of *Bacillus amyloquefaciens* and *P. fluorescence* has accumulated leaf carotenoid content leading to increased leaf chlorophyll content and photosynthetic capacity of fungal-infected *Wilthania somnifera* (Mishra *et al*. 2018).

The overall total phenolic content was generally higher in bioformulation-and benomyl-treated plants compared to Foc-TR4 and control treatments (Fig. 4C). Among benomyl and bioformulation treatments, the phenolic content of talc powder (88.98 μg/g FW) and alginate beads (93.85 μg/g) treatments was the highest; followed by benomyl (64.93 μg/g FW), pesta granules (65.48 μg/g FW) and liquid formulation (49.58 μg/g FW). The Foc-TR4 treated plants contained the lowest phenolic content (38.64 μg/g FW). BCAs have been implicated to induce host production of antimicrobial phenolic compounds via the phenylpropanoid pathway. Phenylalanine was catabolised by the host enzyme phenylalanine ammonia-lyase (PAL) into cinnamic acid which was then converted enzymatically into other phenolic derivatives (Kulbat 2016). A myriad of phenolics such as flavonoids, phytoalexins, lignin and salicylic acid were well-known to possess antifungal properties and they served as the basal defense against phytopathogens (Kulbat 2016; Rais *et al*. 2017). The accumulation of phenolics have contributed to the reduced discolouration in roots, corms and leaf wilting as observed in Foc-TR4 inoculated Berangan plants which was in agreement with previous studies (Ting *et al*. 2004; Fortunato *et al*. 2012; Chen *et al.* 2018).

The LTGA or lignin content of banana roots after 84 DAI was the least for all bioformulation (3.00 to 15.03 μg/g FW) and benomyl (16.24 μg/g FW) treatments as compared to Foc-TR4 treatment (52.46 μg/g FW) (Fig. 4D). The accumulation of lignin content thickens the plant cell wall to strengthen the physical defense against the penetration of fungal pathogens (Barros *et al*. 2015). BCAs were reported to induce lignification in host plants as a form of induce defense system response against plant pathogens (Kulbat 2016). However, the lignin content in banana plantlets treated with bioformulations was very low. In a similar study, the amount of LTGA in root tissues of bananas was also low after treated with various endophytic BCAs at 49 DAI (Ting *et al*. 2004). In a time-course study, the LTGA content peaked in the first few days and gradually decreased thereafter in Foc-TR4 infected roots after pre-inoculated with BCAs (Fishal *et al*. 2010). In other words, BCAs reported in these studies were still able to reduce disease severity though lignification was reduced over a long period of biotic stress. As lignification is the basal defense against plant pathogens, host plants could have triggered other forms of defense signaling pathways for disease suppression.

The proline content was the highest in talc powder (89.61 μg/g FW) which was followed by benomyl (80.89 μg/g FW) and liquid formulation (68.65 μg/g FW) treatments. Pesta granules (56.42 μg/g FW) and alginate beads (54.63 μg/g FW) contained lower proline content than other formulations (Fig. 4E). The lowest proline content was estimated in Foc-TR4 (28.65 μg/g FW) and control treatments (23.15 μg/g FW). Increased proline content was observed in bananas treated with dry or liquid formulations. Several reports have demonstrated that proline accumulation was high in *Fusarium*-and *Sclerotina*-infected host plants when they were inoculated with a consortium of BCAs (Jain *et al*. 2012; Altinok *et al*. 2013). The exact mechanism of proline in host plant defense against pathogens still remains unclear and should be investigated in future.

The MDA content of bioformulation, benomyl and control treatments were overall lower (ranged from 0.49 Nm/g FW to 1.19 Nm/g FW) than Foc-TR4 treatment (3.66 Nm/g FW) (Fig. 4F). Membrane lipid peroxidation was determined by the MDA content. When cells suffer from oxidative burst during root colonisation by plant pathogens, the rate of cell membrane lipid peroxidation increased thereby disrupting the membrane structure integrity of the root cells (Belkhadi *et al*. 2013). In other words, the extent of root cell damage in bioformulation and benomyl treatments was lower than Foc-TR4 treatment. The MDA content was also significantly reduced in *Fusarium*-infected tomato plants after treatments with BCAs (Ferraz *et al*. 2014; Zehra *et al*. 2017)

## CONCLUSION

In general, the application of pesta granules containing a consortium of *P. aeruginosa* DRB1 and *T. harzianum* CBF2 has improved the overall growth in Foc-TR4 infected bananas compared to other bioformulations. The application of bioformulations has also induced host biochemical changes which could have contributed to the suppression of Foc-TR4 under glasshouse condition as reported in our previous study. The use of bioformulations containing BCA consortia is therefore a promising method to manage Foc-TR4 in a sustainable way. To better understand the interaction between BCAs and the banana hosts, DRB1 and CBF2 should be applied simultaneously in Foc-TR4 inoculated and non-inoculated plants. Such observation could allow the validation of the PGP traits and biocontrol efficacy of BCAs. Moreover, field trial should be conducted in future to evaluate the fruit yield and quality of Foc-TR4 infected bananas in order to investigate the effects of DRB1 and CBF2 in promoting fruit reproduction.

## Figures and Tables

**Table 1:** Treatments utilised in glasshouse experiment.

Treatment	Description
Control	Distilled water
Foc-TR4	Spore suspension of Foc-TR4
Benomyl	Benomyl + Foc-TR4
Pesta granules	BCAs + Foc-TR4
Talc powder	BCAs + Foc-TR4
Alginate beads	BCAs + Foc-TR4
Liquid formulation	BCAs + Foc-TR4

**Table 2:** PGP of DRB1 and CBF2.

Isolates	PGP traits				

	P solubilisation (mg/L)	aNitrogen fixation	b,cAmmonia production	b,cSiderophore	IAA (μg/mL)
DRB1	33.7 ± 5.5^b^	√	+ +	+ +	4.1 ± 0.2^a^
CBF2	2273.0 ± 13.4^a^	n.d. d	+ +	+ +	0.0^b^

*Notes*:

a Nitrogen fixation was indicated by ‘√’ whereas ‘−’ indicated no nitrogen fixing activity.

b Biochemical activity was indicated by ‘+’ and ‘−’ signs where ‘−’ = no activity (no halo), ‘+’ = weak activity (halo size ≤ 10 mm), ‘+ +’ = strong activity (halo size ≥ 11 mm and ≤ 20 mm) and ‘+ + +’ = extremely strong activity (halo size ≥ 21 mm).

c Ammonia production was indicated by ‘−’ = no ammonia produced, ‘+’ = slight ammonia produced (yellow), ‘+ +’ = strong ammonia production (dark yellow) and ‘+ + +’ = extremely strong ammonia production (brown).

d n.d. − not determined. Fungi, in nature, do not fix nitrogen.
